# Massachusetts Veterinary Association

**Published:** 1891-06

**Authors:** Austin Peters

**Affiliations:** Secretary


The meeting then adjourned.

M. W. Drake, *Secretary.*

---

*Massachusetts Veterinary Association.*—The annual meeting of the Massachusetts Veterinary Association, was held at Young's Hotel, Boston, Wednesday evening, April 22, 1891.

President Thomas Blackwood, in the chair. Minutes of last meeting read and accepted. Annual reports of the secretary and treasurer read and accepted. The secretary was instructed to continue and subscribe for the Veterinary Journal. Motion made by Dr. Saunders, and seconded by Dr. Hitchings, that the chair appoint a nominating committee to report a list of offlcers for the ensuing year. Carried.

The following nominating committee was then appointed :—Drs. Winchester, Saunders and Hitchings. It reported the following names :—For President, L. H. Howard, D. V. S.; for Vice-President, W. H. Hitchings, D. V. S.; for second Vice-President, W. A. Sherman, D. V. S., M. D.; for Secretary and Treasurer, Austin Peters, M. R. C. V. S.; for Executive Committee, A. Marshall, M. R. C. V. S.; C. E. Hadcock, M. D. V.; E. C. Beckett, M. D. V.; Charles Winslow, D. V. S.; and George Penniman, D. V. S.

Moved by Dr. Winchester and seconded by Dr. Saunders, that the secretary cast one ballot for the list of names appointed by the Nominating Committee. Carried. The secretary then cast one ballot in the affirmative, and the above list of officers were declared duly elected. The new President, Dr. L. H. Howard, then took the chair. Moved by Dr. Hitchings, seconded by Dr. Saunders, that the retiring officers be given a vote of thanks for their services during the past year. Carried unanimously. The meeting then adjourned to the annual dinner.

Austin Peters, *Secretary.*

